# A pregnant woman with a history of SLE with PPROM and severe thrombocytopenia: A case study

**DOI:** 10.1002/ccr3.4792

**Published:** 2021-09-05

**Authors:** Masoumeh Farahani, Arman Maghoul, Matineh Nirouei, Tina Alinia

**Affiliations:** ^1^ Department of Obstetrics and Gynecologists Alborz University of Medical Sciences Karaj Iran; ^2^ Alborz University of Medical Sciences Karaj Iran; ^3^ School of Medicine Faculty of Medicine Tehran University of Medical Sciences Tehran Iran

**Keywords:** case report, placenta abruption, postpartum hemorrhage, pregnancy, systemic lupus erthematous, thrombocytopenia

## Abstract

This study discusses the management of a pregnant woman with PPROM and a history of lupus. She was found to be severely thrombocytopenic which was unresponsive to prednisolone. During cesarean section, placental abruption was found and postpartum hemorrhage ensued.

## Graphical abstract

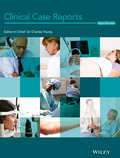

## 1 INTRODUCTION

Systemic Lupus erythematous is a multiple system involvement disease. Lupus has more prevalence in women in reproductive ages. A 41–year‐old woman was referred to emergency department at 32 week of pregnancy because of rupture of membrane. Severe low platelet count was detected in laboratory data; we explained the management of this situation.

Systemic lupus erythematosus (SLE) is a multiple system disease. SLE is characterized by a loss of self‐tolerance, which leads to the activation of autoreactive T and B cells, and in turn, the production of pathogenic autoantibodies and subsequent tissue injury.^1^ Lupus is more prevalent in women (approximately 90% of lupus patients are women) of reproductive age.^2^ It affects one in every 700 women with a peak incidence around the age of 30. The advancements in SLE treatment have made pregnancy possible for more women with this condition; pregnancy loss rate has fallen from 43% in the 1960s to 17% by 2000.^3^ Pregnancy in women with lupus is a high‐risk situation with higher maternal and fetal mortality and morbidity rates in comparison with the general population. The percentage of spontaneous abortions and stillbirths are also higher in women with lupus.^2^ Thrombocytopenia is one of the manifestations of SLE. Thrombocytopenia is defined as a platelet count less than 100,000/mm^3^, and patients are divided into 3 groups according to the severity of their thrombocytopenia: mild (platelet counts >50,000/mm^3^), moderate (>20,000/mm^3^, ≤50,000/mm^3^), and severe (≤20,000/mm^3^).^4^ Severe thrombocytopenia can increase the risk of active bleeding in patients.

## 2 PATIENT INFORMATION

A 41‐year‐old Iranian woman was referred to the emergency department of our hospital at the gestational age of 32 weeks and 5 days of her first pregnancy due to the rupture of membranes, decreased platelet counts, and severe pitting edema in her legs, feet, and ankles. She had a history of systemic lupus erythematosus (SLE), diagnosed at 37 years of age. At that time, she presented with spontaneous petechial rashes and arthralgia and after diagnostic evaluations and tests were done; the diagnosis of SLE was established. Following that, she had no other lupus flare ups. She had been taking azathioprine, prednisolone, and hydroxychloroquine to control her disease and had never discontinued her medications during these years. She had been in remission in the last 4 years. She has a history of right eye cataract. She had never smoked in her life nor had she drank alcohol.

She became pregnant while in remission, and she was closely followed during its course. She had been treated with hydroxychloroquine, azathioprine, and prednisolone. Severely low platelet counts (approximately 9300/mm^3^) were detected in her by means of laboratory testing. She was admitted and treatment was started with prednisolone, azathioprine, and hydroxychloroquine. Her platelet counts did not improve with the admission regimen; therefore, IVIG was added to the adjunct therapy. In addition to thrombocytopenia, anemia (Hemoglobin: 9.9) was also detected; therefore, she received 5 units of platelets and 1 unit of packed red blood cells (pRBCs) in the labor ward.

## 3 CLINICAL FINDINGS

Severely low platelet counts (approximately 9300/mm^3^) were demonstrated in her laboratory data. She also exhibited other signs of lupus flare up such as paresthesia, gingival hemorrhage, musculoskeletal pain, and severe swelling in her legs and feet (other laboratory data are included in Table 1).

**TABLE 1:** Daily Platelet counts in patient

Platelet laboratory data:	/mm^3^
Day 1	9300
Day 2	8000
Day 3	15,000
Day 4	20,200 (Delivery)
Day 5	85,000
Day 6	104,000
Day 7	81,000
Day 8	96,000
Day 9	104,000
Day 10	138,000
Day 11	171,000
Day 12	215,000
Day 13	278,000

The Biophysical Profile Sonography showed a decreased amniotic fluid index (AFI) of 42mm; the overall score was 6/8 (Fetal tone = 2, Fetal activity = 2, Amniotic fluid = 0, Fetal breathing = 2).

## 4 DIAGNOSIS ASSESSMENT

The patient was referred to our hospital due to rupture of membranes and a decreased platelet count and severe pitting edema in the legs, feet, and ankles. She was initially admitted for observation. Laboratory tests were ordered to rule‐out HELLP (Hemolysis, Elevated liver enzyme, Low platelet) syndrome which showed normal liver enzymes and no evidence in favor of hemolysis. Due to the low platelet counts in the initial laboratory reports and the history of SLE, lupus flare up was on top of our differential diagnoses list. Therefore, we used immunosuppressive medication (to control her SLE flare) and tocolytic agents (used for the treatment of preterm labor).

## 5 THERAPEUTIC INTERVENTION

At first, Magnesium sulfate was started to delay labor and 2 doses of betamethasone were administrated during 48 h to precipitate fetal lung maturation. Then, we added ampicillin and erythromycin (for infection prophylaxis), hydroxychloroquine, azathioprine, and prednisolone (to control her SLE flare) as well as calcium‐D to the treatment regimen. Because of decreasing platelet numbers and anemia, we added IVIG to her treatment regimen and she also received 5 units of platelets and 1 unit of packed red blood cell in the labor ward. Her platelet count rose to 20,200/mm^3^ before delivery. Because of decreased fetal movements, increased fetal heart rate (FHR) (170–175/min) and a non‐reactive non‐stress test (NST) after 2 days into her hospitalization, the patient became a candidate for emergency cesarean section (C/S) under general anesthesia with a platelet count of 20,200/mm^3^. Due to the poor response of her platelet count to prednisolone and IVIG, the patient was given 5 more units of platelets and 2 more vials of IVIG (5 grams).

After 1 h into the cesarean section, a preterm baby girl was delivered; she delivered a preterm baby girl (birth weight: 2200 grams, Apgar score: 3/7/9) who was resuscitated and admitted to the neonatal intensive care unit (NICU) with the impression of respiratory distress syndrome (RDS). During surgery, placental abruption was found and postpartum hemorrhage ensued. Intraoperative and postpartum hemorrhage caused approximately 1 liter of blood loss; therefore, a Bakri balloon was fixed and rectal misoprostol was administered.

After the surgery, our patient was admitted to the intensive care unit (ICU) for a duration of 5 days where she received cephalexin, azathioprine, prednisolone, hydroxychloroquine, and enoxaparin. Following an increase in platelet counts (171,000/mm^3^), she was admitted to the ward and stayed under supervision and treatment for 6 days.

## 6 FOLLOW UP AND OUTCOMES

After 13 days of hospitalization, our patient was discharged. Her pitting edema had resolved, and her platelet counts had risen into the normal range (278,000/mm^3^). She had no problem breastfeeding her child. She was asked to come to the clinic after 1 week for follow‐up to ensure lupus quiescence. Her first follow‐up visit showed an improved general condition, and no signs of infection of the incision site were seen. Her platelet counts were within the normal range; she was advised to have routine follow‐ups with her rheumatologist.

## 7 RESULT

In this study, we described a 41‐year‐old pregnant woman with a history of systemic lupus erythematosus who was referred to our hospital due to rupture of membranes at the gestational age of 33 weeks. On laboratory workup, she was found to be severely thrombocytopenic (9300/mm^3^) which was later found to be unresponsive to prednisolone; therefore, IVIG was added to her treatment regimen. On day three of hospitalization, due to decreased fetal movements, increased FHR (170–175/min) and a non‐reactive NST, the patient became a candidate for emergency cesarean section under general anesthesia with a platelet count of 20,200/mm^3^. Since her thrombocytopenia was unresponsive to prednisolone and IVIG, 5 units of platelets were administered before delivery. During the surgery, placental abruption was detected, and postpartum hemorrhage ensued; consequently, a Bakri balloon was fixed and rectal misoprostol was administered. After the cesarean section, she was admitted to the ICU for further medical treatment and supervision.

## 8 DISCUSSION

Pregnancy in an SLE patient creates a dangerous situation for both mother and fetus because lupus can cause maternal death, preeclampsia, preterm labor, thrombosis, infection, and hematologic complications during pregnancy. Thus, managing these patients can be challenging for specialists because they have to create a balance between the mother's treatment and fetal health.^5^, ^6^

Approximately 14% to 26% pregnant women with lupus will develop thrombocytopenia because of increased peripheral platelet destruction, which is induced by antiplatelet antibodies and/or circulating immune complexes.^7^

Two patterns of thrombocytopenia are observed in lupus: a chronic form where no signs of flare ups are present and an acute form which occurs during lupus flares.^8^ Our patient fell into the latter category as she exhibited other signs of lupus flare up such as paresthesia, gingival hemorrhage, musculoskeletal pain, and severe swelling in her legs and feet.

Alkaabi, J.K., et al. published a case report discussing a pregnant woman with severe thrombocytopenia who did not respond to prednisolone or IVIG but responded well to Romiplostim.^9^ The authors believed that thrombopoiesis is regulated by the cytokine thrombopoietin (TPO) binding to its receptor, TPO‐R. When TPO binds to TPO‐R, it activates Janus Kinase 2, which causes megakaryocyte maturation and platelet production. In SLE, autoantibodies against platelet glycoproteins cause a disruption in megakaryocyte maturation culminating in thrombocytopenia.

It has been shown that there are two types of autoantibodies regarding thrombocytes in SLE patients: one against TPO‐R and another against platelet glycoprotein GPIIb/IIIa. The two different autoantibodies have different phenotypes and therapeutic responds. It has been shown that anti‐TPO‐R autoantibodies respond poorly to IVIG; therefore, it is plausible and more likely that our patient developed these types of antibodies.^9^, ^10^

The most challenging aspect of treating lupus during pregnancy is choosing the appropriate medications to treat the mother, considering the fact that those medications could be harmful for the baby.

Lupus treatment regimen in pregnancy consists of hydroxychloroquine, low‐dose steroids, azathioprine and in patients with anti‐phospholipid antibodies, low‐dose aspirin with or without low molecular weight heparin.^11^

Corticosteroids are the main medication used in lupus and are also considered safe during pregnancy. However, use of high doses of corticosteroids during pregnancy is not recommended since it might increase the risk of complications, including diabetes, hypertension, preeclampsia, and premature rupture of membranes.

Hydroxychloroquine should be continued during pregnancy in all patients with SLE. Discontinuation of this drug leads to lupus flares.

Most immunosuppressant agents are contraindicated during pregnancy, but if SLE is in the active phase, azathioprine is considered a safe drug. However, the dose should not exceed 2 mg/kg/day.

During the first and second trimesters, NSAIDs are considered safe medications with no documented increased risk of congenital malformations.

Use of antiplatelet agents during pregnancy is only advised when the maternal benefit clearly outweighs the potential fetal risk.^12^

## CONFLICTS OF INTEREST

The authors declare that they have no conflicts of interest.

## AUTHORS CONTRIBUTIONS

Masoumeh Farahani, Matineh Nirouei, and Arman Mghoul involved in study concept and design. Masoumeh Farahani, Matineh Nirouei, and Arman Mghoul involved in acquisition of data. Matineh Nirouei, Masoumeh Farahani, and Arman Mghoul drafted of the manuscript. Masoumeh Farahani and Tina Alinia involved in critical revision of the manuscript for important intellectual content. Matineh Nirouei, Masoumeh Farahani, and Arman Mghoul involved in administrative, technical, and material support. Masoumeh Farahani involved in study supervision.

## ETHICAL APPROVAL

Informed consent was obtained from the patient for the publication of this case report.

## PATIENT PROSPECTIVE

The prognosis of SLE in pregnancy was discussed with the patient and her husband. It was explained that SLE is a progressive disease and can affect the patient's life on a daily basis.

## CONSENT

A written informed consent was obtained from the patient.

## Data Availability

Data openly available in a public repository that issues datasets with DOIs.
